# NAMPT/Visfatin expression by inflammatory monocytes mediates arthritis pathogenesis by promoting IL-17–producing T cells

**DOI:** 10.1186/1479-5876-10-S3-P48

**Published:** 2012-11-28

**Authors:** Jessy Présumey, Gabriel Courties, Pascale Louis-Plence, Virginie Escriou, Daniel Scherman, Yves-Marie Pers, Hans Yssel, Jérôme Pène, Diego Kyburz, Steffen Gay, Christian Jorgensen, Florence Apparailly

**Affiliations:** 1 INSERM, U844, Montpellier, France; 2 University of Medicine, Montpellier, France; 3 INSERM, U1022, UFR des Sciences Pharmaceutiques et Biologiques, Paris, France; 4 CNRS, UMR8151, Paris, France; 5 University of Pharmacy Paris Descartes, Paris, France; 6 Ecole Nationale Supérieure de Chimie de Paris, Paris, France; 7 University Hospital of Montpellier, Clinical Dept. for osteoarticular diseases, Montpellier, France; 8 Center of Experimental Rheumatology, University Hospital Zurich and Zurich Center of Integrative Human Physiology, Zurich, Switzerland

## Background

Nicotinamide phosphoribosyltransferase (NAMPT)/PBEF/Visfatin exerts multiple functions and has been implicated in the pathogenesis of rheumatoid arthritis. The expression of NAMPT is increased during inflammation and is identified as a novel mediator of innate immunity. To gain insight into its role in arthritis and given that NAMPT induces IL-6 expression that is critical for Th17 lymphocytes, we hypothesized that NAMPT-stimulated production of IL-6 by monocytes might in turn promote Th17 cells.

## Materials and methods

siRNA uptake and NAMPT expression were determined in Ly6C^high^ and Ly6C^low^ monocyte subsets following intravenous injection of siRNA against NAMPT (siNAMPT) or non-targeting siRNA (siCT) formulated with the DMAPAP cationic liposome into mice. Mice with established collagen-induced arthritis (CIA) were treated weekly after disease onset with siNAMPT or siCT and clinical features were assessed. T helper cell frequencies, cytokine production and percentage of IL-6-producing Ly6C^high^ monocytes were analyzed. Using a coculture system consisting of purified CD14^+^ monocytes and autologous CD4^+^ T cells, NAMPT and cytokine production, as well as the percentage of IL-17-producing CD4^+^ T cells were determined following transfection of CD14^+^ monocytes with siCT or siNAMPT.

## Results

Upon intravenous injection, siRNA was preferentially engulfed by Ly6C^high^ monocytes and siRNA-mediated silencing of NAMPT expression in Ly6C^high^ monocytes reduced IL-6 production by these cells, mitigated Th17 cell expansion, and inhibited inflammatory features and CIA progression. Moreover, NAMPT-RNAi-silenced CD14^+^ monocytes were found to reduce the percentage of IL-17-producing CD4^+^ T cells.

## Conclusions

Taken together, our results show that the expression of NAMPT in Ly6C^high^ monocytes promotes Th17 cells. Our findings provide new mechanistic insight into the action of NAMPT in arthritis and demonstrate the utility of targeting disease-causing genes in Ly6C^high^ monocytes for therapeutic intervention in arthritis.

